# Epinephrine Released During Traumatic Events May Strengthen Contextual Fear Memory Through Increased Hippocampus mRNA Expression of *Nr4a* Transcription Factors

**DOI:** 10.3389/fnmol.2018.00334

**Published:** 2018-09-25

**Authors:** Ana Oliveira, Raquel Martinho, Paula Serrão, Mónica Moreira-Rodrigues

**Affiliations:** ^1^Laboratory of General Physiology, Institute of Biomedical Sciences Abel Salazar, University of Porto (ICBAS/UP), Porto, Portugal; ^2^Center for Drug Discovery and Innovative Medicines, University of Porto (MedInUP), Porto, Portugal; ^3^Department of Pharmacology and Therapeutics, Faculty of Medicine, University of Porto (FMUP), Porto, Portugal

**Keywords:** epinephrine, contextual fear memory, traumatic experience, β_2_-adrenoceptors, *Nr4a* transcription factors, phenylethanolamine-*N*-methyltransferase-knockout mice

## Abstract

Epinephrine (EPI) strengthens contextual fear memories by acting on peripheral β_2_-adrenoceptors. Phenylethanolamine-*N*-methyltransferase-knockout (Pnmt-KO) mice are EPI-deficient mice and have reduced contextual fear learning. Our aim was to evaluate the molecular mechanisms by which peripheral EPI strengthens contextual fear memory and if a β_2_-adrenoceptor antagonist can erase contextual fear memories. Pnmt-KO and wild-type (WT) mice were submitted to fear conditioning (FC) procedure after treatment with EPI, norepinephrine (NE), EPI plus ICI 118,551 (selective β_2_-adrenoceptor antagonist), ICI 118,551 or vehicle (NaCl 0.9%). Catecholamines were separated and quantified by high performance liquid chromatography-electrochemical detection (HPLC-ED). Blood glucose was measured by coulometry. Real-time polymerase chain reaction (qPCR) was used to evaluate mRNA expression of nuclear receptor 4a1 (*Nr4a1*), *Nr4a2* and *Nr4a3* in hippocampus samples. In WT mice, plasma EPI concentration was significantly higher after fear acquisition (FA) compared with mice without the test. NE did not increase in plasma after FA and did not strengthen contextual fear memory, contrary to EPI. Freezing induced by EPI was blocked by ICI 118,551 in Pnmt-KO mice. In WT mice, ICI 118,551 blocked blood glucose release into the bloodstream after FA and decreased contextual fear memory. *Nr4a1*, *Nr4a2* and *Nr4a3* mRNA expression decreased in Pnmt-KO mice compared with WT mice after FC procedure. In Pnmt-KO mice, EPI induced an increase in mRNA expression of *Nr4a2* compared to vehicle. In conclusion, EPI increases in plasma after an aversive experience, possibly improving long-term and old memories, by acting on peripheral β_2_-adrenoceptors. Glucose could be the mediator of peripheral EPI in the central nervous system, inducing the expression of *Nr4a* transcription factor genes involved in consolidation of contextual fear memories.

## Introduction

During and after a traumatic experience, fear is a natural reaction. The fear and stress responses enable the organism to react, being responsible for “fight-or-flight” response (Fuller et al., 2010). This response is important for survival and adaptation in nature. Fear conditioning (FC) is a good paradigm to study emotional memories (Quillfeldt, 2016). In this behavioral experiment, animals learn to associate an aversive stimulus (electric shock) to a specific context (Pearce and Hall, 1980). This procedure can be used to study the re-experiencing phenomenon upon exposure to reminders of the trauma (but not the traumatic event itself; VanElzakker et al., 2014).

Stress leads to activation of the sympathetic nervous system which results in an increase of heart rate, contractility, vasoconstriction and release of hormones to bloodstream (Ayada et al., 2015). It has been suggested in some studies that epinephrine (EPI) is an important hormone for memory consolidation in animals and human subjects (Cahill and Alkire, 2003; Dornelles et al., 2007). Furthermore, we also have shown that mice deficient in EPI (phenylethanolamine-*N*-methyltransferase-knockout, Pnmt-KO mice) have reduced contextual fear learning (Toth et al., 2013; Alves et al., 2016). In addition, we found that peripheral EPI strengthens contextual fear memory 1 day after fear acquisition (FA), by acting specifically on peripheral β_2_-adrenoceptors (Alves et al., 2016).

However, EPI is a hydrophilic hormone and does not cross the blood-brain barrier (Weil-Malherbe et al., 1959). One of the hypotheses is that glucose may mediate EPI action in the central nervous system (Morris and Gold, 2013). Indeed, we showed that after FA, wild-type (WT) mice had higher glycemic variation than Pnmt-KO mice, possibly due to EPI release in WT mice after the traumatic event (Alves et al., 2016). EPI may induce liver cells to release glucose into the bloodstream (Hall and Gold, 1986, 1992) and glucose may lead to a subsequent increase of the energy source in the central nervous system and may enhance contextual fear learning (McNay and Gold, 2002; Kong et al., 2017). Downstream molecular processes seem to be a requirement for consolidation and persistence of long-term memories and may result in synaptic remodeling (Dudai, 2002; de la Fuente et al., 2015). Nuclear receptor 4a (*Nr4a*) is a family of transcription factors that has been widely implicated in fear learning and memory (Hawk and Abel, 2011; McNulty et al., 2012). Hawk and Abel showed an increase of the expression of the *Nr4a* family genes in the hippocampus after contextual fear learning (Hawk et al., 2012).

The present study focuses in understanding the mechanism of EPI in strengthening contextual long-term memory (1 day after FA) and old memories (1 month after FA).

## Materials and Methods

### Animals

All animal care and experimental protocols were carried out in accordance with European Directive number 63/2010/EU, transposed to Portuguese legislation by Directive Law 113/2013, and approved by the Organism Responsible for Animal Welfare of Faculty of Medicine, University of Porto. The Pnmt-KO mice (Pnmt^−/–^) were produced by the insertion of Cre-recombinase gene into the locus encoding for Pnmt enzyme, creating a functional KO of Pnmt expression, with loss of EPI in homozygous Pnmt^−/–^. Steven N. Ebert kindly provided a couple of Pnmt-KO mice and animals were bred in our conventional vivarium. Genotypes at the Pnmt locus were identified by polymerase chain reaction (PCR) of ear DNA, as previously described (Ebert et al., 2004). Pnmt-KO (*n* = 92) and WT (*n* = 70) male mice (129×1/SvJ) were kept under controlled environmental conditions (12 h light/dark cycle, room temperature 23 ± 1°C, humidity 50%, autoclaved drinking water, mice diet 4RF25/I and 4RF21/A; Mucedola, Porto, Portugal) and housed with the respective litter.

### Fear Conditioning Procedure

The FC procedure was performed as previously described (Lukoyanov and Lukoyanova, 2006; Manceau et al., 2012). The conditioning chamber consisted of a clear Plexiglas box equipped with a metal grid floor, wired to a stimulus generator. The mice behavior was recorded with a digital video camera Sony HDR-CX405 (Sony Corporation, Japan). On the first day (FA; 6 min (min)), mice had a period of 3 min undisturbed followed by a tone (conditioned stimulus: 80 dB; 2.8 kHz) for 20 s that co-terminated with a foot shock (unconditioned stimulus: 2 s; 0.5 mA). Three tone-shock pairings (conditioning trials) were presented at intervals of 40 s. On the second day (context fear test; 8 min, long-term memory) or 1 month later (context fear test; 8 min, old memories), mice were re-exposed to the conditioning chamber with identical contextual features and no shocks or tones were presented (freezing was scored for the duration of the session). Freezing was defined as the absence of movement except for respiration for at least 3 s (Valentinuzzi et al., 1998; Curzon et al., 2009). The percentage of accumulated freezing time was then calculated. Vocalization response was defined as the audible vocalization in response to the shock, and jump response was defined as the removal of at least three paws from the grid floor (Rocinholi et al., 1997). On the first and second day the chambers were cleaned and wiped with 1% acetic acid.

### Behavioral Treatments

**Figure 1:**
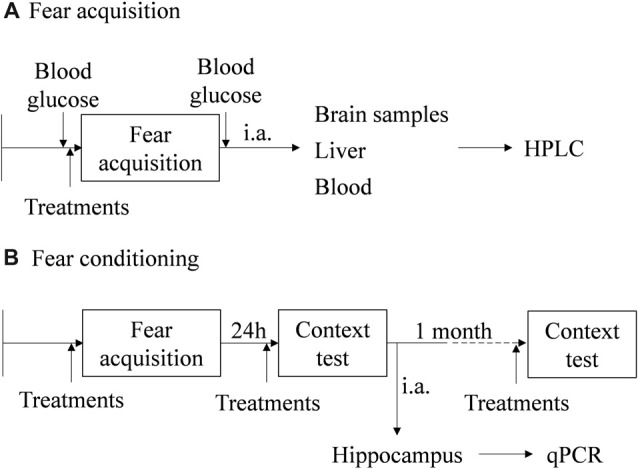
Schematic representation of the behavioral protocols, experimental design, treatments and samples collection. **(A)** Fear acquisition (FA; results in Figures 2, 3). **(B)** Fear conditioning (FC) procedure (results in Figures 4–8). i.a., immediately after; HPLC, high performance liquid chromatography; qPCR, real-time polymerase chain reaction.

Freezing behavior was quantified in WT and Pnmt-KO mice submitted or not to FA. Pnmt-KO mice was submitted to FA after pre-training treatment with EPI (0.1 mg/kg, i.p., 3 min; Lee et al., 2001), ICI 118,551 (2.0 mg/kg, i.p., 30 min; Stone et al., 1996; Zhu et al., 2014) or with vehicle (0.9% NaCl). Another group of WT and Pnmt-KO mice were submitted to FC procedure after EPI (0.1 mg/kg, i.p., 3 min; Lee et al., 2001), norepinephrine (NE, 0.1 mg/Kg, i.p., 3 min; Murchison et al., 2004), EPI (0.1 mg/kg, i.p., 3 min) plus ICI 118,551 (2.0 mg/kg, i.p., 30 min), ICI 118,551 (2.0 mg/kg, i.p., 30 min; Stone et al., 1996; Zhu et al., 2014) or vehicle (0.9% NaCl) treatment, in both pre-training and pre-testing. Long-term memory (context fear test, 1 day after FA) and old memories (context fear test, 1 month after FA) were evaluated. The control groups received the same number of vehicle (0.9% NaCl) injections with the same time interval of the treated groups. A diagram of the experimental design, treatments and samples collection is in Figure 1.

### Catecholamine Assay

Immediately after FA, mice were anesthetized (ketamine, 100 mg/kg and xylazine, 10 mg/kg; i.p.) and the left adrenal was collected and emerged in percloric acid (PCA; 0.2 M) overnight, at 4°C. Afterwards, the supernatant was centrifuged for 2 min (2,700× *g* and 4°C) and diluted. In some experiments blood was collected by left ventricle puncture to a heparinized tube and the samples were centrifuged (15 min, 4°C and 1,000× *g*) and kept under −80 °C until further use. In another experiment, and immediately after FA, liver, hippocampus, amygdala and frontal lobe were collected and emerged in PCA (0.2 M) overnight, at 4°C. The catecholamines present in plasma and tissue samples were concentrated by alumina, as previously described (Moreira-Rodrigues et al., 2010, 2014). Catecholamines were separated by reverse-phase high performance liquid chromatography (HPLC) and quantified by electrochemical detection (ED). The detection limit was between 350 fmol and 1,000 fmol.

### Blood Glucose

Blood glucose concentration was determined immediately before and after the FA in conscious animals in rear paw, by coulometry (FreeStyle Freedom Lite; Alva, 2008). Afterwards, glycemic variation (ΔGlycemia) was calculated as the glucose concentration difference after and before the FA.

### RNA Isolation and Relative Quantification of mRNA Expression

Real-time PCR (qPCR) was performed in hippocampus samples collected immediately after context fear test of FC procedure, as previously described (Moreira-Rodrigues et al., 2007; Mendes et al., 2018). Total RNA isolation was carried out with the SV Total RNA Isolation System kit (Promega, Fitchburg, WI, USA). Concentration and purity of the isolated RNA were measured using the NanoDrop 2000 spectrophotometer (Thermo Scientific, Waltham, MA, USA). Reverse transcription was performed in a T100™ Thermal Cycler (Bio-Rad, Hercules, CA, USA) using a Reverse Transcription kit (Thermo Scientific, Waltham, MA, USA). qPCR reactions were carried out in StepOne™ RT-PCR System (Applied BioSystems, Waltham, MA, USA). Gene-specific primers (5 μM), Maxima SYBR Green qPCR Master Mix (Thermo Scientific, Waltham, MA, USA), Nuclease-free H_2_O (Thermo Scientific, Waltham, MA, USA) were mixed and cDNA was added (1:20). Instead of cDNA, Nuclease-free water (Thermo Scientific, Waltham, MA, USA) was added as a negative control. Gene specific primers are in Table 1. Results of mRNA quantification are expressed in an arbitrary unit (AU) after normalization for Glyceraldehyde 3-phosphate dehydrogenase (GAPDH).

**Table 1:** Primers used in gene expression analysis.

Gene	Primer (5’→3’)
*Nr4a1*	F: AAAATCCCTGGCTTCATTGAG
	R: TTTAGATCGGTATGCCAGGCG
*Nr4a2*	F: CGGTTTCAGAAGTGCCTAGC
	R: TTGCCTGGAACCTGGAATAG
*Nr4a3*	F: GTGGCTCGACTCCATTAAAGAC
	R: GTGCATAGCTCCTCCACTCTCT
*Gapdh*	F: CCATCACCATCTTCGAGGAG
	R: GCATGGACTGTGGTCATGAG

*Nr4, Nuclear receptor 4; Gapdh, Glyceraldehyde 3-phosphate dehydrogenase; F, forward primer and R, reverse primer*.

### Drugs

(−)-EPI (+)-bitartrate salt, L-(-)-NE (+)-bitartrate salt monohydrate, and ICI 118,551 were purchased from Sigma-Aldrich (St. Louis, MO, USA). Ketamine (Imalgene 1000, Merial, Lisboa, Portugal) and xilazine (Rompum 2%, Bayer, Lisboa, Portugal).

### Statistical Analysis

Results are presented as means ± standard error of the means (SEM) for the indicated number of determinations. Catecholamine concentrations, blood glycemic variation and qPCR results were analyzed by unpaired Student’s *t*-test. Behavioral data was analyzed by two-way ANOVA. For multiple comparisons we used Sidak’s test (two groups) or Tukey’s test (three groups). *p* < 0.05 was assumed to denote a significant difference. GraphPad Prism (GraphPad Software Inc., La Jolla, CA, USA) was used for all statistical analysis.

## Results

### Plasma EPI Increased After Fear Acquisition of Fear Conditioning Procedure

**Figure 2:**
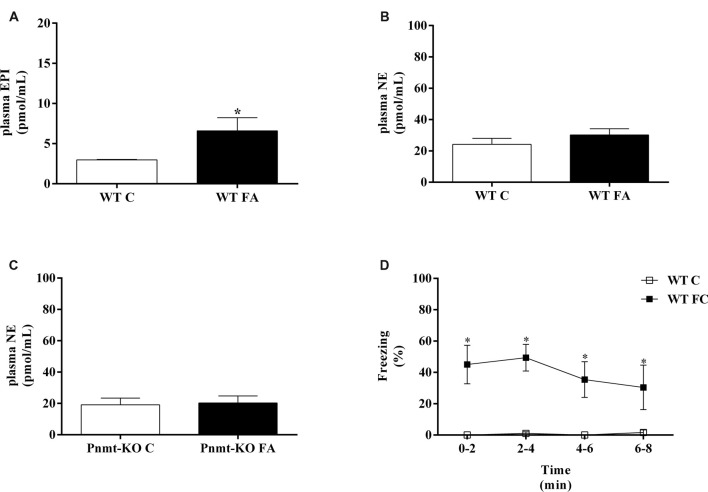
Plasma **(A)** epinephrine (EPI) and **(B,C)** norepinephrine (NE) in wild-type (WT) or phenylethanolamine-*N*-methyltransferase-knockout (Pnmt-KO) mice submitted or not (control, C) to FA of FC procedure. **(D)** Freezing on context fear test in WT mice submitted or not (control, C) to FC procedure. Values are means ± standard error of the means (SEM) of 4–6 mice per group. *Significantly different from correspondent control (C, without FA and FC) values (*p* < 0.05).

In the WT mice, plasma EPI concentration was significantly higher after FA compared with mice without the test (*t*_(8)_ = 4.97, *p* = 0.001; Figure 2A). However, plasma NE concentrations were not different in both WT and Pnmt-KO mice, with or without FA (Figures 2B,C). WT mice submitted to FC procedure showed a significant increase in freezing behavior in FA day of FC procedure compared to WT mice not submitted to FC. It was observed a group (*F*_(1,7)_ = 16.8, *p* < 0.04), time (*F*_(3,21)_ = 21.7, *p* < 0.0001), and a significant interaction between group and time (*F*_(3,21)_ = 20.5, *p* < 0.0001). When re-exposed to the shock context (context fear test) WT mice submitted to FC procedure showed a higher freezing response compared to WT mice not submitted (WT control, Figure 2D). It was observed a group effect (*F*_(1,7)_ = 20.7, *p* = 0.003; Figure 2D). EPI in plasma and in left adrenal glands (Table 2) of Pnmt-KO mice were undetectable. Adrenal NE was significantly increased in Pnmt-KO mice compared to WT mice in both control and FA groups (Table 2). WT mice with or without FA did not present any significant differences in adrenal gland content of EPI or NE (Table 2).

**Table 2:** Concentration of adrenal catecholamines in phenylethanolamine-*N*-methyltransferase-knockout (Pnmt-KO) or wild-type (WT) mice after FA of fear conditioning (FC) procedure.

	EPI (pmol/mL)		NE (pmol/mL)	
	C	FA	C	FA
WT	19.42 ± 3.50	16.91 ± 3.24	8.86 ± 1.73	8.34 ± 2.07
Pnmt-KO	Undetectable	Undetectable	17.42 ± 1.93*	16.85 ± 3.05*

Values are means ± standard error of the means (SEM) of 5–6 mice per group. C, control mice without fear acquisition test; FA, fear acquisition; EPI, epinephrine; NE, norepinephrine. *Significantly different from correspondent values in WT mice (*p* < 0.05).

### EPI Does Not Seem to Cross the Blood Brain Barrier Into the Brain Tissue After Fear Acquisition of Fear Conditioning Procedure

**Figure 3:**
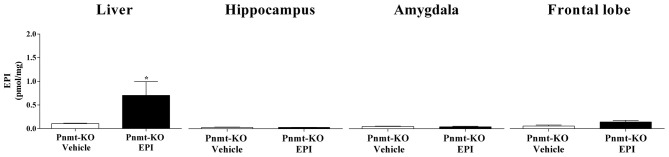
Concentration of EPI in liver, hippocampus, amygdala and frontal lobe after FA of FC procedure in Pnmt-KO mice treated with EPI (0.1 mg/kg) or vehicle (0.9% NaCl). Values are means ± SEM of five mice per group. *Significantly different from correspondent values in Pnmt-KO mice treated with vehicle (*p* < 0.05). EPI content in both treatments of cerebral tissues and in vehicle treatment in liver samples correspond to baseline levels of HPLC technique.

Pnmt-KO mice treated with EPI (0.1 mg/Kg; i.p.) submitted to FA showed a significant increase of EPI in plasma (61.4 ± 9.3 vs. 1.0 ± 0.1 pmol/mL; *p* = 0.0002) and liver (Figure 3) compared to Pnmt-KO treated with vehicle. Furthermore, no differences were observed in EPI levels in hippocampus, amygdala and frontal lobe (Figure 3).

### Intraperitoneal Injection of NE Does Not Increase Contextual Fear Memory

**Figure 4:**
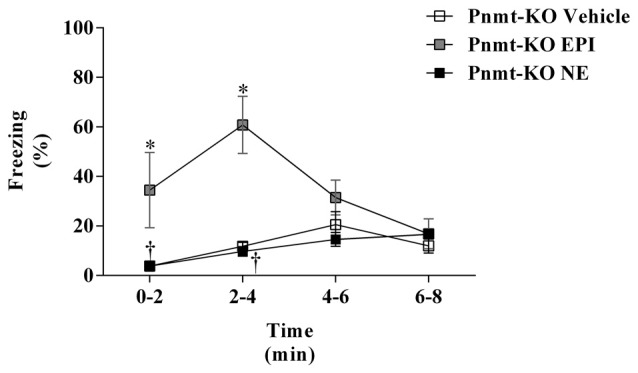
Freezing on the second day (context fear test) of FC procedure in Pnmt-KO mice treated with EPI (0.1 mg/kg), NE (0.1 mg/kg) or vehicle (NaCl 0.9%). Values are means ± SEM of 6–10 mice per group. *Significantly different from correspondent values in Pnmt-KO mice treated with vehicle (*p* < 0.05). ^†^Significantly different from correspondent values in Pnmt-KO mice treated with EPI (*p* < 0.05).

On the first day of FC test, no differences were observed between treatments in Pnmt-KO mice. It was only observed a time effect (*F*_(3,45)_ = 90.9, *p* = 0.0001). On the second day of FC procedure, mice were re-exposed to the shock context which induced an increase in freezing in Pnmt-KO mice treated with EPI (0.1 mg/Kg; i.p.) compared to vehicle (Figure 4). However, no differences were observed in freezing behavior between Pnmt-KO mice treated with NE (0.1 mg/Kg; i.p.) and vehicle (Figure 4). It was observed an effect of drug (*F*_(2,14)_ = 16.5, *p* = 0.0002), time (*F*_(3,42)_ = 4.0, *p* = 0.01) and a significant interaction between drug and time (*F*_(6,42)_ = 4.6, *p* = 0.001; Figure 4).

### EPI Seems to Strengthen Long-Term and Old Memories Through Activation of β_2_-Adrenoceptors

**Figure 5:**
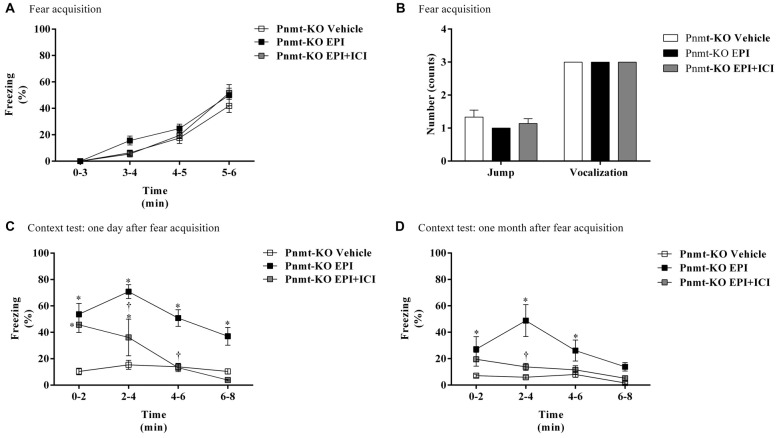
**(A)** Freezing, **(B)** shock responsivity in FA day, and freezing in context fear test **(C)** 1 day and **(D)** 1 month after FA of FC procedure in Pnmt-KO mice treated with EPI (0.1 mg/kg), EPI plus ICI 118,551 (ICI, selective β_2_-adrenoceptor antagonist, 2.0 mg/kg) or vehicle (NaCl 0.9%). Values are means ± SEM of 5–10 mice per group. *Significantly different from correspondent values in Pnmt-KO mice treated with vehicle (*p* < 0.05). ^†^Significantly different from correspondent values in Pnmt-KO mice treated with EPI (*p* < 0.05).

On the first day of FC test, there were no differences in freezing response between treatments (Figure 5A). It was only observed a time effect (*F*_(3,45)_ = 122.7, *p* < 0.0001). No differences were observed in shock responsivity (vocalization and jump) between treatments (Figure 5B). One day after FA, re-exposure to the shock context induced an increase in freezing in Pnmt-KO mice treated with EPI compared to vehicle and EPI plus ICI 118,551 (selective β_2_-adrenoceptor antagonist) treated mice. It was observed an effect of drug (*F*_(2,24)_ = 45.0, *p* < 0.0001), time (*F*_(3,72)_ = 12.3, *p* < 0.0001), and a significant interaction between drug and time (*F*_(6,72)_ = 4.1, *p* = 0.001; Figure 5C). One month after FA, Pnmt-KO mice treated with EPI still showed a significant freezing compared to the other groups. A significant effect of drug (*F*_(2,15)_ = 11.4, *p* = 0.001), time (*F*_(3,45)_ = 8.0, *p* = 0.0002), and an interaction (*F*_(6,45)_ = 3.4, *p* = 0.008) was observed (Figure 5D).

**Figure 6:**
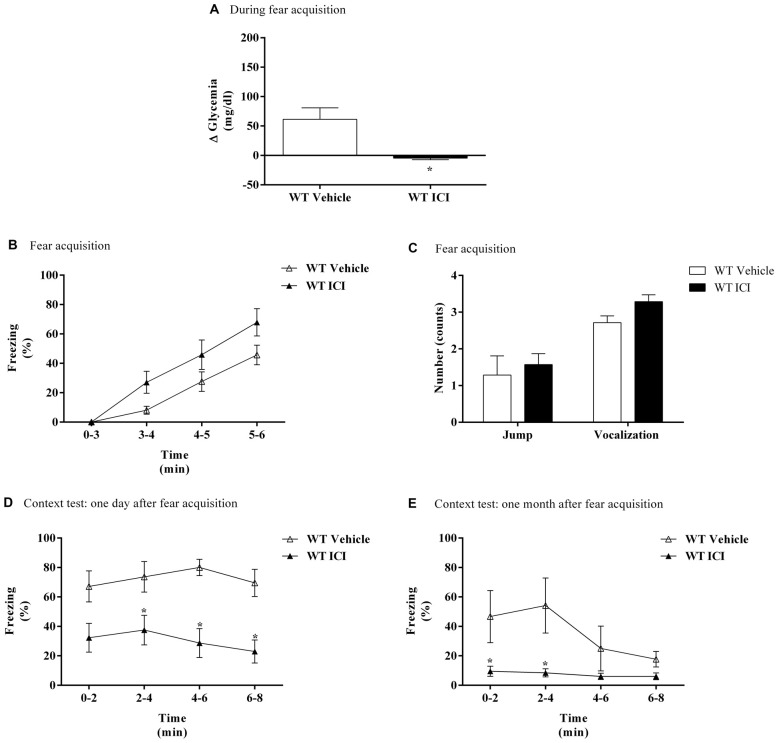
**(A)** Glycemic variation (Δ Glycemia) after FA, **(B)** freezing, **(C)** shock responsivity in FA day, and freezing in context fear test **(D)** 1 day and **(E)** 1 month after FA of FC procedure in WT mice treated with ICI 118,551 (ICI, selective β_2_-adrenoceptor antagonist, 2.0 mg/kg) or vehicle (NaCl 0.9%). Values are means ± SEM of 6–8 mice per group. *Significantly different from correspondent values in WT mice treated with vehicle (*p* < 0.05).

In WT mice, treatment with ICI 118,551 caused a significantly decrease in blood glycemic variation compared with WT mice treated with vehicle after FA (Figure 6A). On the first day of FC test no differences were observed in freezing response between treatments (Figure 6B). It was only observed a time effect (*F*_(3,39)_ = 49.17, *p* < 0.0001). No differences were observed in shock responsivity (vocalization and jump) between treatments (Figure 6C). In context fear test, after re-exposure to shock context the freezing was lower in WT mice treated with ICI 118,551 when compared with WT mice treated with vehicle, in both 1 day and 1 month after FA (Figures 6D,E). It was observed a drug effect in both 1 day (*F*_(1,10)_ = 14.1, *p* = 0.004) and 1 month after FA (*F*_(1,9)_ = 10.1, *p* = 0.01; Figures 6D,E). It was also observed a time (*F*_(3,27)_ = 4.9, *p* = 0.007) effect and an interaction (*F*_(3,27)_ = 3.4, *p* = 0.03) 1 month after FA (Figure 6E).

### Peripheral EPI Appears to Enhance Gene Expression in Hippocampus Involved in Consolidation of Contextual Fear Memories

**Figure 7:**
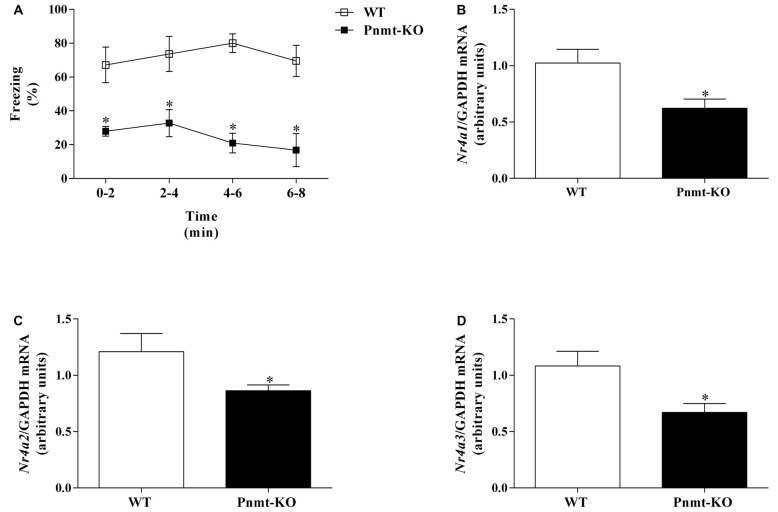
**(A)** Freezing on context fear test and hippocampus mRNA expression of nuclear receptor 4 (Nr4), namely, **(B)** *Nr4a1*, **(C)** *Nr4a2* and **(D)** *Nr4a3* after context fear test of FC procedure in Pnmt-KO and WT mice. Values are means ± SEM of six mice per group. Results of mRNA are expressed as arbitrary units (AUs) after normalization for Glyceraldehyde 3-phosphate dehydrogenase (GAPDH). *Significantly different from correspondent values in WT mice (*p* < 0.05).

In FA day no differences were observed in freezing response between groups. It was only observed a time effect (*F*_(3,36)_ = 59.65, *p* < 0.0001). One day after FA, Pnmt-KO mice showed a lower freezing behavior compared to WT mice after re-exposure to the shock context (Figure 7A). It was observed a genotype (*F*_(1,9)_ = 27.5, *p* = 0.0005) effect (Figure 7A). In the hippocampus, mRNA expression of *Nr4a1* (Figure 7B), *Nr4a2* (Figure 7C) and *Nr4a3* (Figure 7D) was significantly decreased in Pnmt-KO mice compared to WT mice.

**Figure 8:**
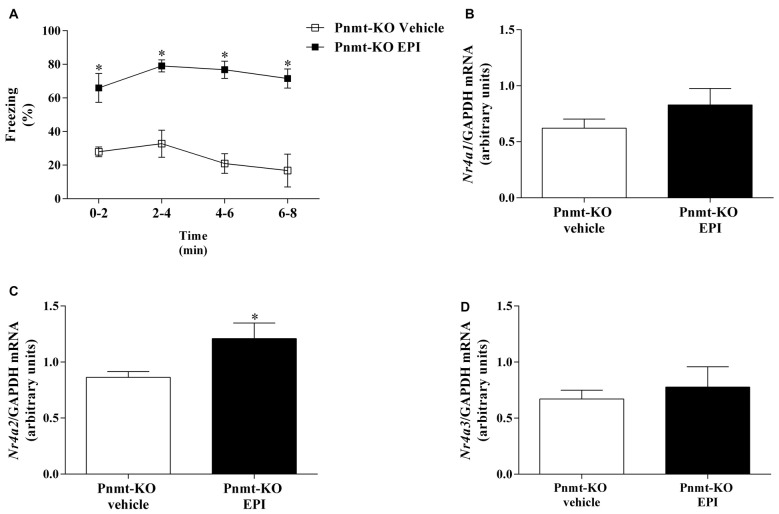
**(A)** Freezing on contextual fear test and hippocampus mRNA expression of Nr4, namely, **(B)** *Nr4a1*. **(C)** *Nr4a2* and **(D)** *Nr4a3* after context fear test of FC procedure in Pnmt-KO mice treated with EPI (0.1 mg/kg) or vehicle (0.9% NaCl). Values are means ± SEM of 6–7 mice per group. Results of mRNA are expressed as AUs after normalization for Gapdh. *Significantly different from correspondent values in Pnmt-KO mice treated with vehicle (*p* < 0.05).

On the first day of FC test no differences were observed in freezing response between treatments. It was only observed a time effect (*F*_(3,33)_ = 128.2, *p* < 0.0001). One day after FA, re-exposure to shock context induced an increase in freezing in Pnmt-KO mice treated with EPI compared to vehicle treated mice (Figure 8A). It was observed a drug effect (*F*_(1,11)_ = 48.2, *p* < 0.0001; Figure 8A). In the hippocampus, mRNA expression of *Nr4a2* was significantly increased in Pnmt-KO mice treated with EPI compared to Pnmt-KO mice treated with vehicle (Figure 8C). No differences were observed in mRNA expression of *Nr4a1* (Figure 8B) and *Nr4a3* (Figure 8D) between groups.

## Discussion

In WT mice the aversive stimulus was sufficient to induce an increase of EPI plasmatic concentration compared to those not submitted to FA. However, plasma NE did not increase in the same conditions. In addition, the increase of plasmatic EPI after FA was accompanied by a strengthening in contextual fear memory compared to those not submitted to FC procedure. Taking together, these results suggest that after stress EPI is released to the bloodstream, possibly from adrenal medulla, and facilitates the formation of emotional contextual fear memories. Accordingly, in mice, i.p. injections of EPI (0.1 mg/kg, i.p.) facilitate retention in inhibitory avoidance test (Introini-Collison et al., 1992). We now show that i.p. injections of EPI strengthen contextual fear memory, contrary to i.p. injections of NE, in similar doses. It appears that peripheral NE is not important for contextual fear memory, opposing the known effects of central NE in memory (McGaugh, 2002; Tully and Bolshakov, 2010).

Furthermore, it was shown that dopamine β-hydroxylase KO mice (deficient in both NE and EPI; Murchison et al., 2004) and later Pnmt-KO mice (deficient in EPI; Toth et al., 2013; Alves et al., 2016) have reduced contextual fear learning. In addition, we found that EPI strengthens contextual fear learning on long-term memory (1 day after FA), by acting specifically on peripheral β_2_-adrenoceptors (Alves et al., 2016). Our results show that pre-training and pre-testing treatments with ICI 118,551, a β_2_-adrenoceptor antagonist, decreases long-term memory (1 day after FA) and possibly 1-month old fear memories. Thus, EPI seems to be an important mediator of long-term consolidation of fear memory and even old memories.

EPI is a polar molecule that does not cross the blood-brain barrier (Weil-Malherbe et al., 1959) and Pnmt has almost no expression and enzymatic activity in the brain (Ho et al., 1974; Lew et al., 1977). However, there are some evidences that intense stressful situations can lead to physiological changes in blood-brain barrier permeability (Skultétyová et al., 1998; Sántha et al., 2016) and consequently in molecules that normally do not cross the blood-brain barrier (as is the case of EPI) might cross (Hayes et al., 1985). For that reason, we performed an experiment to understand if EPI could cross the blood-brain barrier to the brain tissue after our stress conditions using an EPI deficient (Pnmt-KO) mice. Pnmt-KO mice were submitted to FA and EPI was found in plasma and liver samples in mice injected i.p. with this molecule, but not in Pnmt-KO treated with vehicle. These results indicate that EPI (i.p.) goes into the bloodstream and reaches the hepatic tissue of EPI deficient (Pnmt-KO) mice. However, we found that after FA (stress event), Pnmt-KO mice treated with EPI did not present this catecholamine in many brain regions, namely hippocampus, amygdala, and frontal lobe. Therefore, our results show that EPI does not appear to cross the blood-brain barrier after the acute stress event in our experimental conditions.

In FA (first day of FC procedure) we did not observe any differences in freezing, vocalization and jump responses between treatments, in both Pnmt-KO and WT mice. Our results suggest that pain perception of the foot shocks is not affected by treatments. These results are in agreement with previous manuscripts (Toth et al., 2013; Alves et al., 2016).

In response to an increase of plasmatic EPI concentration, glycogenolysis and gluconeogenesis processes are stimulated and hepatic glucose production increases (Gray et al., 1980; Dufour et al., 2009). We already showed that i.p. injections of EPI induces an increase in glycemic variation in Pnmt-KO mice, suggesting that glucose may be a downstream mediator of EPI in long-term memory formation and a critical component of fear memory modulation (Alves et al., 2016). Furthermore, we now show in WT mice that i.p. injections of ICI 118,551 (peripheral β_2_-adrenoceptor antagonist) is effective in blocking the action of endogenous EPI, not occurring an increase of blood glucose levels nor contextual fear memory formation. Thus, because glucose easily crosses the blood-brain barrier, we emphasize that it can be a mediator of EPI in the central nervous system, since the increase of blood glucose levels may provide additional energy for specific memory mechanisms, leading to an increase of contextual fear learning (Durkin et al., 1992; Pych et al., 2005).

Intensive research suggests that memory consolidation occurs within minutes to weeks after initial learning and may reflect the ongoing changes in the intracellular signaling pathways, gene expression, and new protein synthesis by leading to synaptic modifications and gene plasticity (Dudai, 2002). As we already showed, Pnmt-KO mice have a decrease of contextual fear memory in comparison to WT mice (Alves et al., 2016), and we now show a significant decrease in hippocampus mRNA expression of the three members of *Nr4a* family (*Nr4a1*, *Nr4a2* and *Nr4a3*). This suggests that essential molecular pathways for contextual fear memory formation are not activated due to the absence of EPI release after a traumatic event, and possibly the lack of glucose availability in the brain as the energy source for neuronal mechanisms. When we restored EPI levels with i.p. injections in Pnmt-KO mice, we found an increase in contextual fear memory and a significant increase only in *Nr4a2* mRNA expression in Pnmt-KO mice. This could suggest that the *Nr4a2* gene expression may be sufficient to strengthen contextual fear memory by peripheral EPI. *Nr4a* transcription factors family is increased 2 h after acquisition (Vecsey et al., 2007) and are necessary to contextual fear memory formation (Hawk et al., 2012). We show that *Nr4a* gene family continues upregulated 24 h after fear learning, which suggests their requirement for long-lasting memory storage. Actually, *Nr4a* gene family may function in memory to activate secondary waves of transcription (Vecsey et al., 2007).

Previous reports indicate that the lack of EPI is enough to mimic the impairment of contextual fear learning in the context-dependent conditioned fear, without affecting cue-dependent conditioned fear (Toth et al., 2013; Alves et al., 2016). As EPI, glucose also improves retention of contextual fear learning (but not cued learning; Glenn et al., 2014; Alves et al., 2016). Neuron cells that are under direct control of glucose availability, that appear to be specific to hippocampus (de Araujo, 2014) could be the reason for the elevated mRNA expression of *Nr4a* family genes in hippocampus after FC procedure in mice with peripheral EPI (WT and Pnmt-KO treated with EPI). Our and other results are a strong evidence that EPI presence in a stressful situation leads to an increase of blood glycemia (Sutherland et al., 1968). In addition, the increase of mRNA expression of *Nr4a2* in hippocampus only occurs in mice with EPI (WT mice and in Pnmt-KO mice treated with EPI).

In conclusion, EPI increases in plasma after an aversive situation which may cause an increase in blood glucose by acting in peripheral β_2_-adrenoceptors. In turn, glucose may be a mediator of EPI in the central nervous system and could be essential to induce the expression of the *Nr4a* family genes involved in contextual fear memories. β_2_-adrenoceptor antagonists decrease long-term contextual fear memory and possibly old memories.

## Author Contributions

MM-R conceived the study. AO and MM-R performed most of the experiments. RM and PS performed some experiments. AO, MM-R and PS analyzed and interpreted the results and performed the statistical analysis. AO and MM-R wrote the manuscript. MM-R and RM revised the manuscript. All authors have reviewed and approved the final manuscript.

## Conflict of Interest Statement

The authors declare that the research was conducted in the absence of any commercial or financial relationships that could be construed as a potential conflict of interest.
